# A Rare Case of Adult Non-traumatic Recurrent Anterior Hip Joint Dislocation

**DOI:** 10.7759/cureus.106402

**Published:** 2026-04-03

**Authors:** Fuminori Kamakura, Yoshihito Tsuzaki, Tomohito Matsushita, Yoshimasa Ishigaki, Gaku Yasuda

**Affiliations:** 1 Department of Orthopedic Surgery, Fujimi-Kogen Hospital, Fujimi-Kogen Medical Center, Fujimi, JPN; 2 Department of Internal Medicine, Fujimi-Kogen Hospital, Fujimi-Kogen Medical Center, Fujimi, JPN; 3 Department of Radiology, Fujimi-Kogen Hospital, Fujimi-Kogen Medical Center, Fujimi, JPN

**Keywords:** adult, anterior hip dislocation, corticosteroid, myopathy, non-traumatic, recurrent

## Abstract

A 75-year-old woman, who could walk independently with a walker, was incidentally found to have anterior hip joint dislocation on computed tomography. She had a history of anti-signal recognition particle immune-mediated necrotizing myopathy (SRP IMNM), for which long-term oral corticosteroid treatment was administered. Closed reduction was performed immediately after hip dislocation diagnosis; however, the joint easily dislocated again anteriorly. The patient could walk independently with the dislocated joint and did not wish to undergo further treatment. The findings from this case, together with those from previous reports, suggest that long-term corticosteroid administration may be a risk factor for non-traumatic hip dislocation. The underlying myopathy involving significant muscle atrophy may have played a critical role in the pathogenesis of this joint instability.

## Introduction

Most cases of hip dislocation result from high-energy trauma, whereas non-traumatic dislocations are extremely rare [1]. Generally, anterior hip dislocation accounts for a small percentage of all cases because of the robust joint capsule, strong ligamentous structures, and specific mechanisms required for such an injury [2]. Reported risk factors for non-traumatic dislocation include disorders involving pelvic dysmorphism or soft-tissue fragility [3-6]; however, reports of pharmacotherapy-related risk factors are scarce. Long-term corticosteroid use inhibits collagen synthesis and increases the degradation of connective tissue components, leading to generalized ligamentous laxity and capsule thinning. This pharmacological effect can significantly compromise the structural integrity of the primary stabilizers of the hip joint.

In this report, we present a case of non-traumatic anterior hip dislocation discovered incidentally in a patient receiving long-term corticosteroid therapy for anti-signal recognition particle (SRP) antibody-positive immune-mediated necrotizing myopathy (IMNM). This case proposes a potential clinical hypothesis regarding pharmacotherapy-related risk factors for non-traumatic hip dislocations. Specifically, the findings indicate that prolonged steroid administration may induce fragility in joint-supporting tissues, warranting further investigation as a potential risk factor for non-traumatic dislocation.

## Case presentation

**Figure 1:**
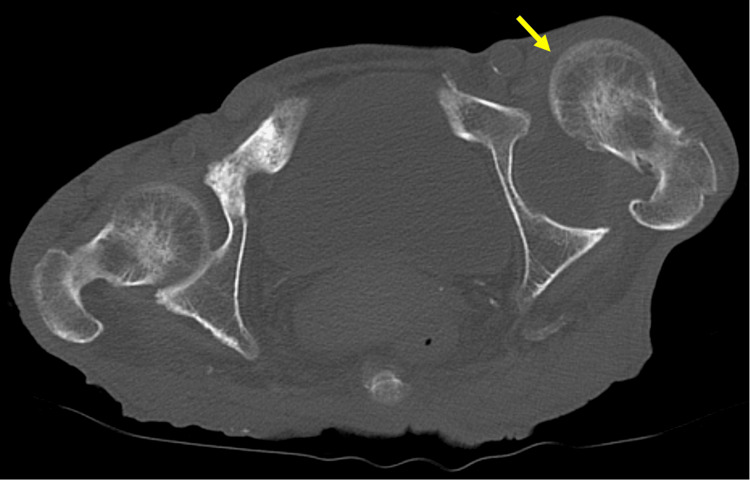
First computed tomography (CT) scan. The first CT scan incidentally revealed anterior dislocation of the left hip joint.

A 75-year-old woman, who could walk independently with a walker, was referred to our orthopedic department. She had undergone a computed tomography (CT) scan in the internal medicine department for the evaluation of abdominal symptoms and malignancy screening, which incidentally revealed an anterior hip joint dislocation (Figure 1). In detailed history taking, neither the patient nor her family reported any preceding hip pain, instability, or changes in her baseline gait prior to the CT examination.

**Figure 2:**
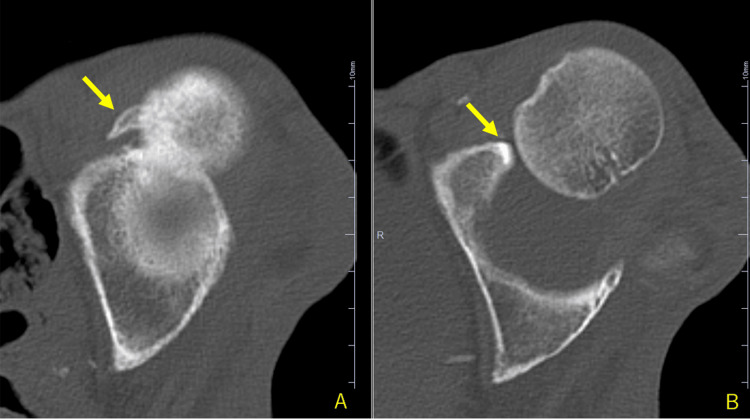
First CT scan of the left hip joint. A beak-shaped osteophyte is visible on the posterior side of the femoral head (A), along with bone wear on the anterior rim of the acetabulum (B).

A beak-shaped osteophyte on the posterior side of the femoral head was observed on the CT scan (Figure 2A), along with bone wear on the anterior rim of the acetabulum (Figure 2B).

The patient reported that the dislocation occurred without any inducement. She had a history of anti-SRP IMNM and had been receiving oral corticosteroids for at least 25 years. Prednisolone was the primary corticosteroid administered throughout the clinical course. The total cumulative exposure was approximately 75,000 mg, with a maximum dose of 50 mg/day. The maintenance dose was approximately 30 mg/day during the first year, which was subsequently tapered and maintained at 5-15 mg/day. Regarding other therapies, intravenous immunoglobulin (IVIg) was administered during the initial phase of the disease. During the maintenance phase, the treatment regimen included periods of administration of tacrolimus, methotrexate, and azathioprine.

**Figure 3:**
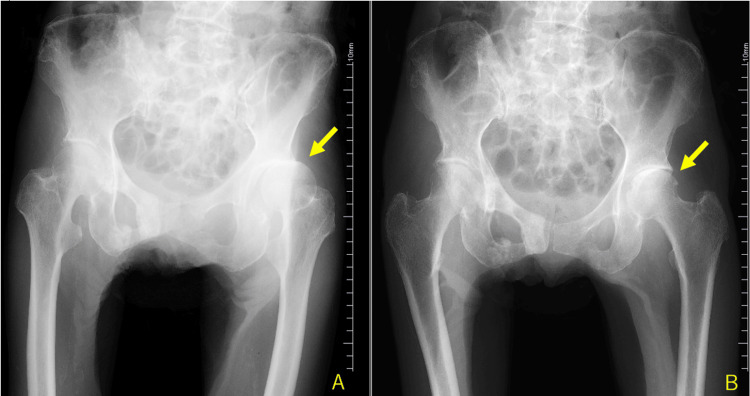
Plain radiographs of the hip joint. The dislocated left hip joint (A) was reduced very easily by internal rotation (B).

Closed reduction was performed immediately after diagnosis of the dislocation. Given that the dislocated joint (Figure 3A) was reduced with only minimal internal rotation force, the reduction procedure was easily completed without any anesthesia (Figure 3B). Evaluation of stability after reduction revealed that the joint remained stable and did not dislocate again, except that external rotation force was applied.

**Figure 4:**
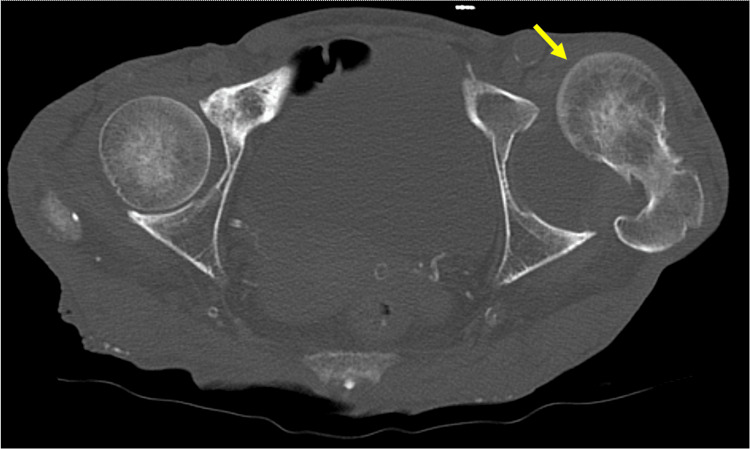
CT scan right after the reduction. The left hip joint redislocated without any external force.

Subsequently, while maintaining the supine position, the hip joint spontaneously dislocated again anteriorly during the patient’s transfer to the CT room (Figure 4), even without any application of external force or external rotation.

**Figure 5:**
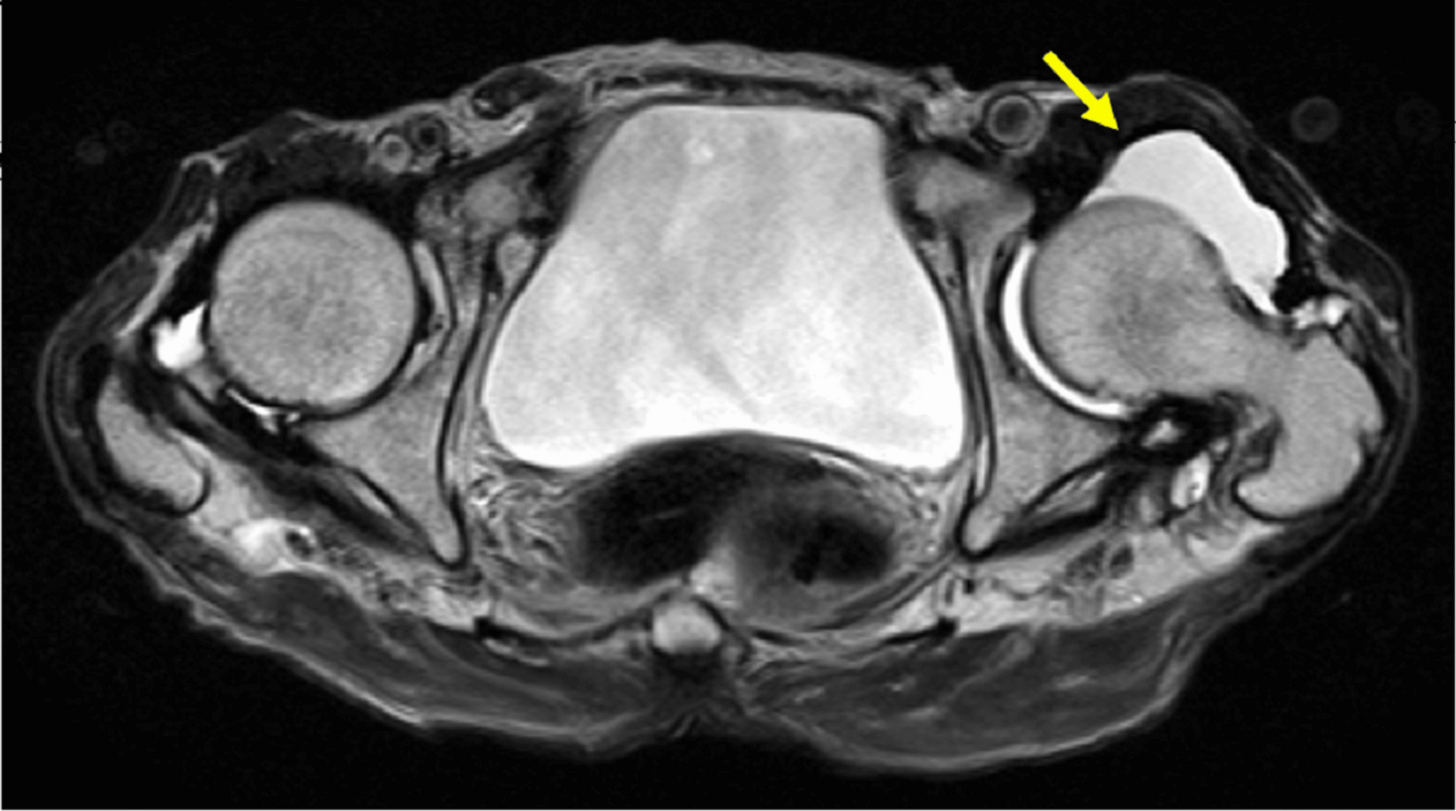
Magnetic resonance imaging (MRI) of the hip joint. T2-weighted MRI showing a small amount of joint effusion, rupture of the joint capsule, marked atrophy of the gluteus muscles, thinning of the iliopsoas tendon, and loosening of the soft tissue on the anterior side of the hip joint.

Further evaluation of the magnetic resonance scan revealed a small amount of joint effusion, rupture of the joint capsule, marked atrophy of the gluteus muscles, and thinning of the iliopsoas tendon (Figure 5). These findings suggest a global fragility of the dynamic and static stabilizers of the hip joint.

Despite the dislocated joint, the patient could walk independently without difficulty using a walker, and no further treatment was administered following her preference. The patient provided informed consent for the publication of this report and any accompanying images.

## Discussion

Most hip dislocations result from high-energy trauma, with non-traumatic dislocations being extremely rare [1]. The frequency of anterior traumatic hip dislocation is low, at only 7-13 % [2]. Anterior dislocations are rare because abduction and external rotation are unusual injuries [1]. The ligamentous structure of the anterior joint capsule tissue is strong, which makes anterior dislocation less likely [1]. Abnormal pelvic morphology, soft tissue abnormalities, hip joint hypoplasia, acetabular fracture, infection, paralysis, Down syndrome, Ehlers-Danlos syndrome, and von Recklinghausen syndrome have been reported as contributing factors [3-6]. Excessive debridement of the iliopsoas tendon, acetabular labrum, and ligamentum teres has been reported in iatrogenic hip dislocation after arthroscopy [7].

In the present case, anterior dislocation of the hip joint occurred without any particular induction and was considered non-traumatic. Only a few case reports have described non-traumatic hip dislocations. Fischer et al. [10] reported a case of non-traumatic bilateral recurrent atraumatic hip dislocation in a patient with asthma, and Trousdale [3] reported a case of non-traumatic hip dislocation in a patient with systemic lupus erythematosus; both patients were receiving long-term oral corticosteroids. Our patient had anti-SRP IMNM and had been receiving long-term oral corticosteroid treatment for several decades. These observations suggest that long-term corticosteroid administration contributes to non-traumatic hip dislocation. In addition, the underlying IMNM must be considered a major contributing factor. The chronic nature of anti-SRP IMNM often results in profound atrophy of the pelvic girdle muscles [8]. This loss of muscular support, alongside steroid-induced collagen synthesis inhibition in the joint capsule [9], likely created a multifactorial predisposition to dislocation. Several previously reported cases of non-traumatic hip dislocations have been on the anterior side of the joint; although anterior dislocations are rare in traumatic dislocations, this may be a characteristic of non-traumatic dislocations [3,10].

Liebenberg and Dommisse [11] suggested that one possible mechanism of recurrent hip dislocation is increased hydrostatic pressure within the joint capsule, causing the ruptured portion of the capsule to act as a one-way valve, resulting in pseudobursa dislocation. In the present case, joint effusion and rupture of the anterior joint capsule were observed, suggesting that this mechanism may have been involved. In addition, in the present case, CT imaging revealed specific bony changes (Figure 2). We believe that the beak-shaped osteophyte on the posterior aspect of the femoral head, positioned in proximity to the anterior acetabular rim, resulted from the chronic dislocated state. Similarly, given that the patient maintained independent ambulation despite the dislocation, the bone wear on the anterior acetabular rim was likely a secondary change due to the chronic weight-bearing and joint instability.

Prompt reduction of the dislocation is necessary to improve symptoms and avoid avascular necrosis, cartilage damage, and compression of nearby nerves and blood vessels [12]. Rotational acetabular osteotomy is performed as a surgical treatment [4]. In the present case, the patient did not report any symptoms. Although the risks mentioned above were considered, the patient and her family opted for conservative management.

This case report has certain limitations that merit consideration. First, as a single-case study, a causal relationship between corticosteroid use and joint capsule rupture could not be definitively established. In addition, while the patient reported no specific trauma, the possibility of minor, forgotten low-energy movements (e.g., during sleep or transition) contributing to the initial displacement cannot be entirely excluded. Finally, the absence of any histopathological examination of the joint capsule limits our understanding of the specific cellular changes induced by long-term steroid therapy in this patient.

## Conclusions

Herein, we report a rare case of incidentally diagnosed non-traumatic anterior hip dislocation. We suspect that tissue fragility due to long-term steroid administration may have contributed to the dislocation in this case. Accordingly, we propose that increased clinical awareness regarding potential joint instability may be warranted in patients receiving long-term steroid therapy, particularly those with additional risk factors, such as periarticular muscle atrophy and underlying myopathy. Future research should focus on prospective observational studies of joint laxity in patients receiving long-term steroid therapy. Additionally, investigating whether specific myopathies, such as anti-SRP IMNM, may independently contribute to joint instability by inducing periarticular muscle atrophy remains a pertinent area for future investigation.
